# The contribution of work characteristics, home characteristics and gender to burnout in medical residents

**DOI:** 10.1007/s10459-016-9710-9

**Published:** 2016-09-20

**Authors:** Hanne Verweij, Frank M. M. A. van der Heijden, Madelon L. M. van Hooff, Jelle T. Prins, Antoine L. M. Lagro-Janssen, Hiske van Ravesteijn, Anne E. M. Speckens

**Affiliations:** 1 Department of Psychiatry, Radboud university medical center, PO Box 9101, 6500 HB Nijmegen, The Netherlands; 2 Department of Neuropsychiatry, Vincent van Gogh Institute for Psychiatry, Venlo, The Netherlands; 3 Behavioural Science Institute, Radboud University, Nijmegen, The Netherlands; 4 MCL Academy, Medical Center Leeuwarden, Leeuwarden, The Netherlands; 5 Department of Primary and Community Care, Unit Gender and Womens’ Health, Radboud university medical center, Nijmegen, The Netherlands

**Keywords:** Burnout, Gender, Job demands and resources, Home demands and resources, Medical residents

## Abstract

Burnout is highly prevalent in medical residents. In order to prevent or reduce burnout in medical residents, we should gain a better understanding of contributing and protective factors of burnout. Therefore we examined the associations of job demands and resources, home demands and resources, and work–home interferences with burnout in male and female medical residents. This study was conducted on a nation-wide sample of medical residents. In 2005, all Dutch medical residents (n = 5245) received a self-report questionnaire on burnout, job and home demands and resources and work–home interference. Path analysis was used to examine the associations between job and home characteristics and work–home interference and burnout in both males and females. In total, 2115 (41.1 %) residents completed the questionnaire. In both sexes emotional demands at work and the interference between work and home were important contributors to burnout, especially when work interferes with home life. Opportunities for job development appeared to be an important protective factor. Other contributing and protective factors were different for male and female residents. In females, social support from family or partner seemed protective against burnout. In males, social support from colleagues and participation in decision-making at work seemed important. Effectively handling emotional demands at work, dealing with the interference between work and home, and having opportunities for job development are the most essential factors which should be addressed. However it is important to take gender differences into consideration when implementing preventive or therapeutic interventions for burnout in medical residents.

## Introduction

Burnout is highly prevalent in medical residents. Depending on the specialty 27–75 % of residents suffer from burnout (Ishak et al. 2009). In the Netherlands, approximately one-fifth of medical residents indicate to have moderate to severe burnout symptoms (Prins et al. 2010). Burnout is defined as a syndrome of emotional exhaustion, depersonalization and a diminished sense of personal accomplishment. Emotional exhaustion refers to the feelings of being exhausted and physically overextended; energy is lacking and mood is low. Depersonalization is characterized by feelings of cynicism and detachment toward patients. Reduced personal accomplishment is marked by a tendency to evaluate oneself negatively, particularly with regard to work with patients (Demerouti et al. 2001; Maslach and Jackson 1986). Burnout may lead to less work satisfaction, disrupted personal relationships, substance abuse, depression, and even suicide (De Valk and Oostrom 2007; van der Heijden et al. 2008). Interventions to decrease burnout in medical residents are scarce and only a few studies have been conducted. Ripp et al. (2015) recently found that the implementation of duty hours restrictions did not change burnout rates in American internal medicine residents.

A recent meta analysis indicated that interventions including psycho education, interpersonal communication, and mindfulness meditation may results in decreased burnout levels in physicians (Regehr et al. 2014). In order to improve interventions to prevent or reduce burnout in medical residents, we should gain a better understanding of the contributing and protective factors of burnout.

The job demands–resources model (JD-R model) is an often used model to explain burnout and (dis)engagement at work. The model assumes that demands at work lead to constant overtaxing and a lack of resources complicates the meeting of job demands which can lead to burnout and disengagement (Bakker and Demerouti 2007; Demerouti et al. 2001). However every occupation may have its own specific work characteristics associated with burnout. Furthermore, the JD-R model proposes that the interaction between demands and resources is important for the development of burnout as well (Bakker and Demerouti 2007). Job *demands* refer to work characteristics that require physical and/or psychological effort and are associated with certain physiological and/or psychological costs. Job *resources* refer to those aspects of the job that are functional in achieving work goals, reduce job demands and associated physiological and psychological costs, or stimulate personal growth and development (Bakker et al. 2004). The JD-R model was tested and confirmed in several populations, also in health care professionals (Houkes et al. 2008; Zis et al. 2014).

In the last decade, research indicates that the interference between work and home is also important in burnout (Bakker and Geurts 2004; Bakker et al. 2011; Byron 2005; Demerouti et al. 2010). Work–home interference (WHI) and home–work interference (WHI) can be experienced positively when positive experiences from one role make it easier to enhance participation in the other role, and negatively when pressures from the work and family roles are incompatible, such that participation in one role makes it difficult to participate in the other (Greenhaus and Beutell 1985; Grzywacz and Marks 2000). In American surgeons work–home conflict appear to be a major contributor to burnout, especially for female surgeons (Dyrbye et al. 2011a, b). However, there is still little research on the direct impact of home demands and resources on burnout. A study in a Dutch working population indicated that home demands were associated with burnout (Peeters et al. 2005).

With the increasing influx of women in the medical profession it is very relevant to examine possible gender differences in the relationships between work and home characteristics on the one hand and burnout on the other hand (van der Velden et al. 2008). Prins et al. (2010) indicated that in Dutch medical residents females are more emotionally exhausted but less depersonalized than their male colleagues. Research suggests that the pattern of associations between work and home characteristics and burnout might be different for men and women as well (Houkes et al. 2008; Langballe et al. 2011).

The aim of the present study was to test a model including all the potential contributing factors of burnout together, in order to examine which factors are most important and where interventions could focus on. In addition, we investigated whether there are gender differences with regard to possible contributing or protective factors of burnout. We formulated the following hypotheses:

### **Hypothesis 1**

Model testing: We expect (a) job and home demands to be positively related to burnout, (b) job and home resources to be negatively related to burnout, (c) Job/home resources to buffer the relationship between job/home demands and burnout, (d) negative work–home interference to be positively related to burnout, and positive work–home interference to be negatively related to burnout.

Gender differences: Because it is difficult to a formulate firm hypothesis about the role of gender in the proposed model, in this study we aim to explore the generalizability of the model across male and female medical residents.

## Study context

The study was conducted in the Netherlands, where residency training closely follows undergraduate medical education. The 6 years of undergraduate training lead to the MD degree and a basic qualification to practice medicine. Residency programs are run by university medical centers in close collaboration with affiliated general hospitals and vary in length from 3 to 6 years depending on specialty. Before residency, most doctors work as residents-not-in-training to increase their likelihood of obtaining a post in the residency program of their preference. Residents are allowed to work with a maximum of 48 working hours a week including educational activities, and attendance and accessibility shifts. In the Netherlands, postgraduate medical education reformed in the 2000s focusing on competency-based medical education. Since 2004 these reforms were supported by governmental bodies.

## Methods

### Participants and procedure

This study is conducted on a nation-wide sample of Dutch medical residents, which has been collected in a previous study aimed at gaining insight in the prevalence and levels of burnout and work engagement in medical residents in the Netherlands (Prins et al. 2010). In this study we are interested in the work and home characteristics that are associated with burnout. All 5245 Dutch medical residents in training on October 1, 2005, were invited to take part in the survey. They received a self-report questionnaire at their home address and they could choose to complete the questionnaire anonymously by hand or online. A cover letter explained that purpose of the study and emphasized anonymity. All residents were sent three reminders and a non-response form within a timeframe of 2 months.

### Measures

#### Burnout

Burnout was measured using de validated Dutch version of the Maslach Burnout Inventory-Human Service Survey (MBI-HSS) (Maslach and Jackson 1986; Schaufeli and Van Dierendonck 2000). This measure is considered to be the “gold standard” to measure burnout symptoms and has been found to be reliable and valid (Maslach et al. 2008). The MBI-HSS is specifically developed for healthcare professionals. It consisted of 20 items measuring three burnout subscales emotional exhaustion (8 items, α = .89) depersonalization (5 items, α = .73) and reduced personal accomplishment (7 items, α = .79). Items were scored on a seven-point Likert scale, ranging from (0) ‘never’ to (6) ‘always’. Example item: “I feel emotionally drained from my work” (emotional exhaustion); “I don’t really care what happens to some patients” (depersonalization); and “I deal very effectively with the problems of my patients” (reduced personal accomplishment).

#### Job demands

Workload (4 items, α = .87), emotional demands at work (6 items, α = .79) and mental demands at work (4 items, α = .77) were each measured by shortened scales of the Questionnaire on the Experience and Evaluation of Work (QEEW) (Veldhoven and Meijman 1994). Participants responded on a five-point scale from (1) ‘‘never’’ to (5) ‘‘always.’’ Example items: “Do you have to work very fast?” (workload); “Is your work emotionally demanding?” (emotional demands), and “Does your work demand a lot of concentration?” (cognitive demands).

#### Job resources

Six job resources were measured using scales developed by Bakker et al. (2004): Job autonomy (3 items, α = .73), job development (3 items, α = .80), social support from colleagues (3 items, α = .84), performance feedback (5 items, α = .83), supervisory coaching (6 items, α = .86), participation in decision making (4 items, α = .77). All items were scored on a 5-point rating scale ranging from 1 (“never/poor/totally disagree”) to 5 (“always/good/totally agree”). Example items: “Do you have freedom in carrying out your work activities?” (autonomy); “At work I am given the opportunity to develop my personal strengths.” (job development); “Can you, when necessary, ask your colleagues for help.” (social support from colleagues); “I receive enough feedback from my supervisor in regards to my work.” (performance feedback); “My supervisor uses his/her influence to help me solve my problems at work.” (supervisory coaching); and “I feel that I am involved in making important decisions” (participation in decision making).

#### Home demands

Three home demands were measured using scales developed by Peeters et al. (2005): Home workload (5 items, α = .75), emotional demands (3 items, α = .76) and mental demands (3 items, α = .88). All items were scored on a 5-point rating scale ranging from 1 (“never”) to 5 (“always”). Example items: “Do you have to carry out a lot of tasks at home [household/caring tasks]?” (home workload); “Are you confronted with situations in your private life that are emotionally charged??” (emotional demands); and “Do you have to plan and organize a lot of things in relation to your home life?” (mental demands).

#### Home resources

Three home resources were measured using scales developed by Demerouti et al. (2010): personal autonomy (4 items, α = .82), social support from partner/family (4 items, α = .87) and opportunity for personal development (3 items, α = .88). All these items were scored on a 5-point rating scale ranging from 1 (“never”) to 5 (“always”). Example items: “I manage daily life at home” (personal autonomy); “My family/partner pays attention to my feelings and problems” (social support from partner/family); and ‘‘I can develop my talents during my free time’’ (opportunity for personal development).

*Work*–*home interference* was measured using the ‘‘Survey work–home interaction NijmeGen SWING’’(Geurts et al. 2005): Positive work–home interference (3 items, α = .42), negative work–home interference (3 items, α = .73), positive home–work interference (3 items, α = .68) and negative home–work interference (3 items, α = .79). All these items were scored on a 5-point rating scale ranging from 1 (“never”) to 5 (“always”). Example items: “How often does it happen that after a pleasant working day, you feel more in the mood to engage in activities with your spouse/family/friends?” (positive WHI); “How often do you find it difficult to fulfill your domestic obligations because you are constantly thinking about your work?” (negative WHI); “How often does it happen that after spending a pleasant weekend with your spouse/family/friends, you have more fun in your job?” (positive HWI); and “How often do you do not fully enjoy your work because you worry about your home situation?” (negative HWI).

### Data analysis

We analyzed the data by means of path analysis using the Mplus7 statistical software package (Muthén and Muthén 1998–2012). Path analysis is a subset of Structural equation modelling (SEM) using only measured variables and no latent variables, and is used to examine associations between two or more variables. The associations or pathways in the models represent hypotheses, which are based on previous research and theoretical propositions. First, we examined the associations between work and home demands and resources and work–home interference on the one hand, and the three burnout subscales on the other hand, comparing five models against each other. We chose to include work characteristics in our analyses first, and to add home characteristics in a next step, in order to be able to examine if including home characteristics explained an additional proportion of the variance in burnout, beyond the effects of work characteristics. Secondly, to examine possible gender differences in these associations we employed multi-group path-analysis in Mplus7 (Muthén and Muthén 1998–2012). The (improvement in) fit of the various models in both steps was assessed using the Chi-square difference test, as well as the RMSEA (Root Mean Square Error of Approximation) and the CFI (comparative fit index). Values of .90 and higher (CFI) and .08 or lower (RMSEA) indicate an acceptable fit (Byrne 2013). Because of the large data set and the number of variables included in this model we chose to indicate *p* values below .01 as significant in order to decrease the chance of type 1 errors. To determine the contributing and protective factors of burnout, the standardized path coefficients (Beta values) were calculated. These indicate the patterns of associations between the contributing/protective factors and burnout. Standardized path coefficients (β) with values of less than .10 can be interpreted as small effects, values of around .30 can be interpreted as medium effects and values above .50 can be interpreted as large effects (Kline 1998).

## Results

### Study population

Of the 5245 residents who were invited in October 2005, 105 indicated that they were no longer residents. Of the remaining 5140 residents, 125 (2.4 %) indicated that they did not wish to participate and 2900 (56.4 %) did not respond. In total, 2115 (41.1 %) completed the questionnaire (Prins et al. 2010). Characteristics of the respondents are presented in Table 1. Male residents worked on average 52.3 (6.7) hours per week, female residents 49.6 (7.4) hours per week. Table 2 displays the means and standard deviations of the study variables for both males and females. Female residents experienced more workload at home, emotional demands at home and more negative home–work interference that male residents. But they also experienced more personal autonomy at home and more social support from family/partner. Male residents reported more social support from colleagues, more supervisory coaching and more personal development at home than their female colleagues.

**Table 1:** Characteristics of the respondents (N = 2115)

Variable	Mean (SD)	N	%
Gender			
Female		1290	61.1
Male		820	38.9
Age, range 23–58 years	31.5 (3.5)		
Children <18			
One or more children		663	31.7
No children		1429	68.3
Years in training	3.0 (1.5)		
Medical specialty in groups		2115	
Internal medicine		292	13.8
Other medical specialties		497	23.5
General surgery		170	8.0
Other surgical specialties		354	16.8
Obstetrics and gynaecology		125	5.9
Paediatrics		162	7.7
Psychiatry		242	11.5
Supportive specialties		270	12.8

*SD* standard deviation

**Table 2:** Descriptive statistics by gender

Variable	Total	Females	Males	*p* value
	Mean (SD)	Mean (SD)	Mean (SD)	
*Job demands*				
Workload	3.31 (.80)	3.29 (.80)	3.33 (.79)	.42
Emotional demands	2.47 (.59)	2.46 (.57)	2.47 (.58)	.77
Mental demands	4.00 (.59)	4.01 (.59)	3.97 (.59)	.07
*Job resources*				
Job autonomy	3.04 (.69)	3.02 (.67)	3.06 (.71)	.23
Job development	3.75 (.64)	3.74 (.63)	3.76 (.67)	.44
Social support from colleagues	3.61 (.86)	3.56 (.87)	3.67 (.84)	.004^b^
Performance feedback	3.10 (.75)	3.08 (.74)	3.14 (.76)	.08
Supervisory coaching	2.86 (.75)	2.82 (.74)	2.93 (.75)	.003^b^
Participation in decision-making	3.03 (.75)	3.01 (.74)	3.06 (.77)	.139
*Home demands*				
Workload	3.12 (.74)	3.21 (.75)	2.99 (.70)	<.001^a^
Emotional demands	2.29 (.73)	2.31 (.75)	2.27 (.69)	.18
Mental demands	2.65 (1.00)	2.69 (1.05)	2.59 (.92)	.02^c^
*Home resources*				
Personal autonomy	3.44 (.77)	3.57 (.76)	3.25 (.75)	<.001^a^
Social support from family/partner	3.64 (.87)	3.69 (.89)	3.57 (.83)	.002^b^
Personal development	2.85 (.91)	2.76 (.92)	2.97 (.88)	<.001^a^
*Work–home interference*				
WHI positive	2.43 (.59)	2.42 (.59)	2.46 (.60)	.11
WHI negative	2.28 (.70)	2.30 (.66)	2.26 (.74)	.25
HWI positive	2.60 (.75)	2.60 (.74)	2.61 (.77)	.73
HWI negative	1.64 (.55)	1.67 (.56)	1.59 (.54)	.001^b^

*SD* standard deviation, *WHI* work–home interference, *HWI* home–work interference

^a^Statistically significant baseline difference between groups at *p* < .001; ^b^ Statistically significant baseline difference between groups at *p* < .01; ^c^ Statistically significant baseline difference between groups at *p* < .05

### General model for burnout

In the first model [M1: χ^2^(111) = 751.33, RMSEA = .06, CFI = .75] job demands and job resources were related to each of the three subscales of burnout. As indicated by the relatively low CFI value, this model did not fit well to the data. Building on this first model, we examined a second model [M2: χ^2^(93) = 500.14, RMSEA = .05, CFI = .84] which also included home demands and home resources. Adding these paths significantly improved the fit of our model [∆χ^2^(18) = 251.19, *p* < .001]. In Model 3 [M3: χ^2^(39) = 426.45, RMSEA = .07, CFI = .85], we extended M2 by including the interactions between work demands and work resources, in order to examine the proposition of the JD-R model that resources mitigate the positive associations between demands and burnout (Bakker and Demerouti 2007). In order to avoid problems of multicollinearity, these interactions were computed using mean-centred scores (Aiken et al. 1991). As M3 did not provide a better fit than M2 [∆χ^2^(54) = 73.69, ns], these interactions were omitted from further analyses. Model 4 [M4: χ^2^(66) = 469.71, RMSEA = .06, CFI = .84] was also based on M2, and additionally included the interactions between home demand and home resources. Compared to M2, this model did not improve model fit either [∆χ^2^(27) = 30.43, ns]. Our final model [M5: χ^2^(81) = 107.58, RMSEA = .01, CFI = .99] was also based on M2 but included also positive and negative home–work and work–home interference. This final model showed a good fit in absolute sense and fitted significantly better than M2 [∆χ^2^(12) = 392.56, *p* < .001]. The path analysis supported hypothesis 1a, 1b and 1d, indicating the relationships between job demands and resources, home demands and resources, and work–home interferences on the one hand and burnout on the other hand. In contrary to hypothesis 1c resources did not mitigate the association between demands and burnout.

### Separate models for burnout in male and female residents

We started by modelling the paths that were included in M5 in female and male residents separately. Initially, we imposed equality constraints on all structural paths, thus assuming the strength of all associations to be similar for both sexes. This model 6 provided a good fit to the data [M6: X^2^(219) = 322.64, RMSEA = .02, CFI = .96]. Subsequently, in a second model [M7: X^2^(162) = 225.36, RMSEA = .02, CFI = .98] we removed these equality constraints to examine if this resulted in a better fitting model. Indeed M7 (without constraints) fitted significantly better than M6 (with constraints) (∆X^2^(57) = 97.28, *p* < .001). Therefore, we concluded that our data provided evidence for gender differences in the associations between work, home and work–home interference characteristics and burnout in medical residents.

### Contributing and protective factors of burnout

**Fig. 1:**
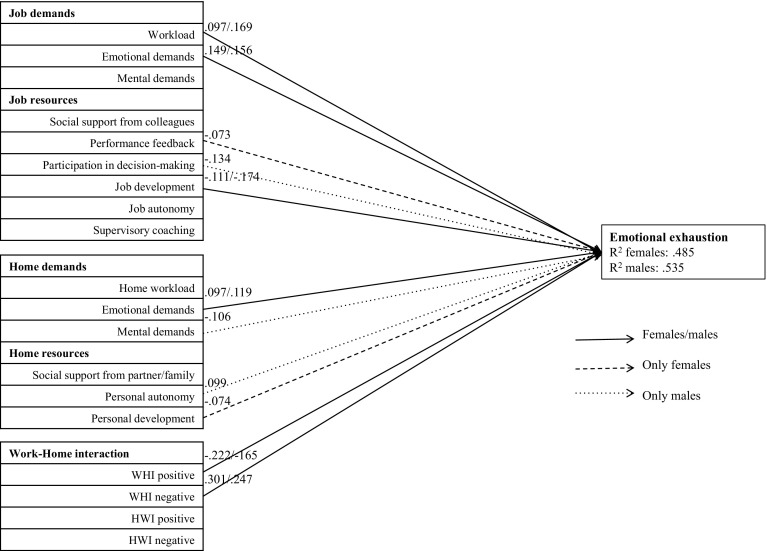
Path analysis of emotional exhaustion. Only associations that were statistically significant at *p* < .01 are presented. *WHI* work–home interference; *HWI* home–work interference

**Fig. 2:**
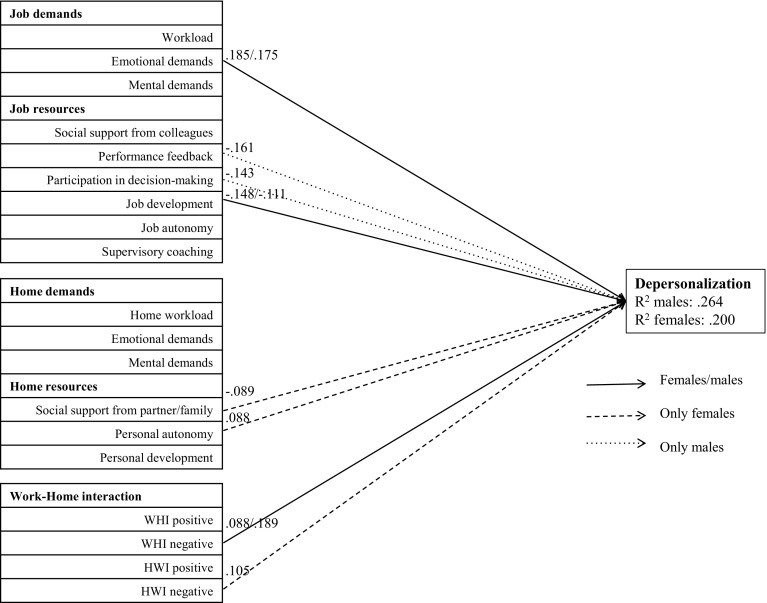
Path analysis of depersonalization. Only associations that were statistically significant at *p* < .01 are presented. *WHI* work–home interference; *HWI* home–work interference

**Fig. 3:**
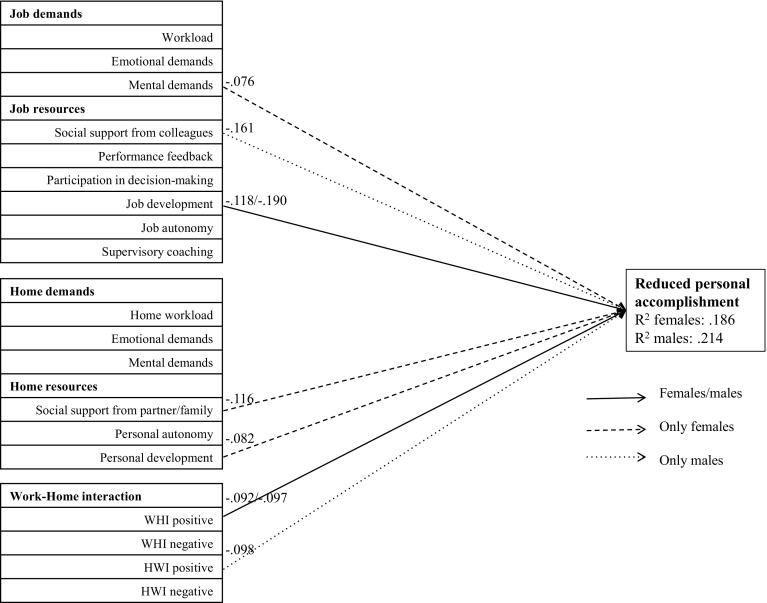
Path analysis of reduced personal accomplishment. Only associations that were statistically significant at *p* < .01 are presented. *WHI* work–home interference; *HWI* home–work interference

Figures 1, 2 and 3 provide graphical representations of the model (divided in the three burnout subscales) and show the significant associations with their standardized path coefficients (Beta values). Because of the significant gender differences, the paths are presented for female and male residents separately.

The factors in this model explain a significant proportion of variance of emotional exhaustion (R^2^ men: .535; R^2^ women: .485), depersonalization (R^2^ men: .264; R^2^ women: .200) and reduced personal accomplishment (R^**2**^ men: .214; R^**2**^ women: .186). All significant contributors and protectors of the three subscales of burnout are described below.

#### Emotional exhaustion

With regard to the significant positive associations with emotional exhaustion, we found that workload and emotional demands at work contributed to emotional exhaustion in both sexes. Emotional demands at home also contributed to emotional exhaustion in both sexes. Surprisingly, in males personal autonomy at home contributed to emotional exhaustion. For both sexes, negative work–home interference contributed to emotional exhaustions.

In addition to contributing factors, we also found several protecting factors of emotional exhaustion. Opportunities for job development was protective against emotional exhaustion in both sexes, possibly more pronounced in males than in females. Participation in decision-making was protective only in males. In females, performance feedback was protective, but this was a relatively small effect. Regarding the home characteristics, personal development at home was a small but significant protecting factor against emotional exhaustion in females. Surprisingly, in male residents, mental demands at home was protective. Positive work–home interference was protective against emotional exhaustion in both sexes.

#### Depersonalization

We found several work characteristics that contributed to depersonalization. Emotional demands contributed to depersonalization in both sexes. In contrast with our expectations, in females personal autonomy appeared contribute to depersonalization to a small but significant extent. Negative work–home interference significantly contributed to depersonalization in females and even more so in males. Negative home–work interference significantly contributed to depersonalization only in females.

With regard to protective factors, opportunities for job development were protective against depersonalization in both sexes. Performance feedback and participation in decision-making were protective only in males. Social support from partner/family was a small but significant protecting factor against depersonalization only in females.

#### Reduced personal accomplishment

There were no job or home characteristics, nor any work–home interferences which contributed to reduced personal accomplishment. We did, however, find several job characteristics that were protective against reduced personal accomplishment. In females, mental demands at work was a small but significant protecting factor against reduced personal accomplishment. Opportunities for job development was protective against reduced personal accomplishment in both sexes but to a greater extent in male residents. Social support from colleagues was protective only in males. With regard to home resources, social support from partner/family and personal development at home were protective against reduced personal accomplishment only in females. In both sexes, positive work–home interference was protective. Positive home–work interference was protective only in males.

## Discussion

The aim of the present study was to examine the associations of work and home characteristics and work–home interference with burnout in Dutch male and female medical residents.

First of all, we examined the differences between males and females in experienced home and work characteristics. We found interesting differences such as males feeling more supported by colleagues than females, and females reporting more negative interferences from home to work than their male colleagues. These results should be interpreted with caution given that these differences might have changed during the last decade. However, the gender differences we found are consistent with more recent findings on job demands and resources and work stress in German physicians, where male physicians reported more job resources than female physicians (Mache et al. 2016). A recent study by Abrahams et al. (2013) in Dutch general practitioners also found women to report more negative home to work interference, and less support from colleagues than men, which indicates that gender differences may still be present.

Secondly, we examined the associations between these characteristics and burnout in males and females. In general, the results provided support for our hypothesized model, we found work characteristics as well as home characteristics and work–home interference to predict burnout. Assessing the predictors in our model in more detail, we found that in both sexes emotional demands rather than workload or mental demands is an important contributing factor to burnout. This is in line with research in human service employees by Vegchel et al. (2004), who indicate that that emotional demands are as important as, and sometimes more important than, quantitative demands. Furthermore, work–home interference was also strongly associated to burnout, which is in line with research from Peeters et al. (2005) and Langballe et al. (2011), who both indicate that work–home interference plays an important role in burnout. In terms of protecting factors, opportunities for job development are important for both male and female residents, which was also found in a recent study among neurology trainees in Greece (Zis et al. 2015). However, some significant gender differences were found in the associations with burnout. For male residents, resources or potentially protective factors tended to be confined mostly to work, while for female residents, resources tended to originate in both the home and the work setting. This is consistent with both older and a more recent studies (Greenglass and Burke 1988; Langballe et al. 2011).

## Strengths and limitations

One of the strengths of this study is the large dataset used to test the model. All medical residents in The Netherlands were invited to participate. This resulted in a large, representative dataset with residents from different parts of the country, different hospital settings and different medical specialties.

The present study also has certain limitations. First of all, the data were collected at the end of 2005 and beginning of 2006. So, some of our findings might have been overhauled by developments in the post-graduate training of medical residents or social or cultural changes that have since taken place. However, this might affect the absolute levels of burnout and its contributors more than their mutual relationships. In 10 years the absolute values of these variables might have been changed but we believe that the relationships between these variables and burnout have not been affected by such changes, as the mechanisms underlying the associations between antecedent variables and burnout are universal and are not expected to change over time. The job demands–resources model, on which we based our hypotheses, assumes that job demands in combination with a lack of resources lead to constant psychological overtaxing and disengagement, and in the long run burnout (Demerouti et al. 2001). There is no reason to believe that this process will have changed during the past decade or that the specific characteristics associated with burnout in medical residents have drastically changed because the nature of the job has not essentially changed. However, it would be interesting to repeat the survey in a few years in order to compare the data.

Secondly, although considering the nature of the survey the response rate was quite high (41 %), more than half of the medical residents who received an invitation for the survey did not respond. This could have led to a selection bias in our sample, because the non-respondent might differ from the responders. Common reasons for not responding in those who took the trouble to send in their non-response form (2.4 %) appeared to be a lack of energy (11 %) and a lack of time (22 %). Individuals who are burned out may be less likely to return questionnaires, which might have resulted in an underestimation of burnout in medical residents in this study. However, again, this might especially have affected the absolute levels of burnout and its contributors instead of their relationships. Third, the generalizability of our findings is limited to medical residents. It is likely that the relevant demands and resources and the influence of gender vary in different occupations. Another limitation is that the data were collected at one point in time, so we were not able to look into possible longitudinal relationships in the model. In addition, the causality could not be established in this study. For instance, it could be that emotional demands at work lead to burnout, but it could also be that people with high levels of burnout may also perceive higher emotional demands at work. However, longitudinal research by Hakanen et al. (2008) did not find evidence for the reversed effect of burnout on job demands and resources. Finally, all measures were based on self-reports thus causing a concern for a common method bias. Ideally, future research should combine self-report measures with more objective measures, such as computerize tasks, observations and records on working hours, absenteeism and long term sickness absence.

## Practical implications and future research

When developing possible interventions to address burnout in medical residents we should take account of the above. First of all, it is important to teach residents how do deal with the emotional demands at work as this seemed an important predictor of burnout. It is often the emotionally demanding aspects of medicine that may also be the most meaningful and satisfying. Although there is awareness of the importance of emotional wellbeing in health care workers, there is still little attention for effectively managing emotions in medical education. The informal curriculum (physician role modelling) and the medical culture still advocates emotional detachment, distance and clinical neutrality (Shapiro 2011, 2013). Intervision in peer groups and supervision or coaching programs during residency should specifically include dealing with emotional demands. Furthermore, interventions or educational approaches that incorporate aspects of allowing, acknowledging and regulating emotions, such as mindfulness training and Balint groups should be stimulated as well (Dobkin and Hutchinson 2013; Epstein 1999; Kjeldmand and Holmström 2008; Perry et al. 2013).

Furthermore, interventions that promote balance in work and home responsibilities should be stimulated and studied as work–home interference was found to be an important predictor of burnout. This could happen by providing facilities such as childcare, flexible working hours and by creating a culture where a balance in work and home is valued and supported by the institutions. In addition to decreasing the impact of potential contributing factors to burnout we should also focus on strengthening the protective factors we found such as opportunities for development, feedback and support. Institutions could stimulate these job resources by providing a good learning climate where residents are inspired, supported and are given constructive feedback by their supervisors so that they can learn and grow.

Although many important factors in burnout were the same in male and female residents, the gender differences that were found should be taken into account. In female residents, home resources were more often protecting factors against burnout than in male residents, while in male residents job resources such as social support from colleagues and participation in decision-making seemed important against burnout. Burnout is not only a work-related issue, home characteristics are also associated with burnout, especially in female residents. This should be taken into consideration when implementing or developing preventive or therapeutic interventions for burnout in medical residents.

This study raises many questions that can only be answered by additional research. Deeper understanding on other potential influences of burnout such as race or ethnicity, social situations and personality characteristics will require far broader investigations. Future research should also involve longitudinal studies to find more support for the predictive nature of the associations found in our model and to understand the mechanisms underlying gender differences in association between demands/resources and burnout. Furthermore, future research should focus on evaluating training programs or interventions to decrease burnout in medical residents.
